# Oxidative Molecular Layer Deposition Tailoring Eco-Mimetic Nanoarchitecture to Manipulate Electromagnetic Attenuation and Self-Powered Energy Conversion

**DOI:** 10.1007/s40820-023-01112-7

**Published:** 2023-05-31

**Authors:** Jin-Cheng Shu, Yan-Lan Zhang, Yong Qin, Mao-Sheng Cao

**Affiliations:** 1 School of Materials Science and Engineering, Beijing Institute of Technology, Beijing, 100081 People’s Republic of China; 2 Institute of Coal Chemistry, State Key Laboratory of Coal Conversion, Chinese Academy of Sciences, 27 Taoyuan South Rd, Taiyuan, 030001 Shanxi People’s Republic of China

**Keywords:** Oxidative molecular layer deposition, Eco-mimetic nanoarchitecture, Microwave absorption, Electromagnetic attenuation, Self-powered energy conversion device

## Abstract

Advanced electromagnetic devices, as the pillars of the intelligent age, are setting off a grand transformation, redefining the structure of society to present pluralism and diversity. However, the bombardment of electromagnetic radiation on society is also increasingly serious along with the growing popularity of "Big Data". Herein, drawing wisdom and inspiration from nature, an eco-mimetic nanoarchitecture is constructed for the first time, highly integrating the advantages of multiple components and structures to exhibit excellent electromagnetic response. Its electromagnetic properties and internal energy conversion can be flexibly regulated by tailoring microstructure with oxidative molecular layer deposition (oMLD), providing a new cognition to frequency-selective microwave absorption. The optimal reflection loss reaches ≈  − 58 dB, and the absorption frequency can be shifted from high frequency to low frequency by increasing the number of oMLD cycles. Meanwhile, a novel electromagnetic absorption surface is designed to enable ultra-wideband absorption, covering almost the entire K and Ka bands. More importantly, an ingenious self-powered device is constructed using the eco-mimetic nanoarchitecture, which can convert electromagnetic radiation into electric energy for recycling. This work offers a new insight into electromagnetic protection and waste energy recycling, presenting a broad application prospect in radar stealth, information communication, aerospace engineering, etc.

**Supplementary Information:**

The online version contains supplementary material available at 10.1007/s40820-023-01112-7.

## Highlights

- Drawing wisdom and inspiration from nature, an eco-mimetic nanoarchitecture is constructed, featuring tunable electromagnetic properties and high-efficiency energy attenuation.
- Through in-depth insight into the microstructure, the material basis of electromagnetic response is clearly revealed to establish an intrinsic connection between microscopic electronic structure and macroscopic electromagnetic properties.
- A creative self-powered energy conversion device is constructed, with the integrated functions including electromagnetic protection and waste energy recycling, which offers a new horizon for the fields of energy and environment.

**Supplementary Information:**

The online version contains supplementary material available at 10.1007/s40820-023-01112-7.

## Graphical abstract

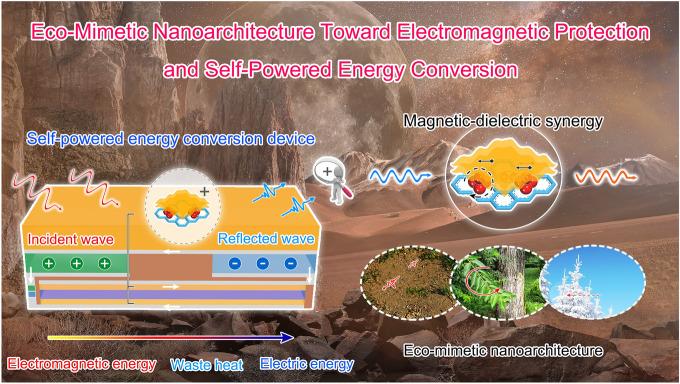

## Introduction

With the rapid development of artificial intelligence and wireless communication technology, human society is facing a drastic transformation, which will profoundly affect traditional industries and life modes [1–3]. Advanced electromagnetic and energy devices as the important pillars of the transformation can effectively promote the development of society toward intelligence, information, and convenience [4–6]. In recent years, great efforts have been devoted to investigating the novel electromagnetic and energy devices, and fruitful results have been achieved [7–10]. Chen's group fabricated honeycombed-like carbon aerogels with embedded Co@C nanoparticles by directionally freeze-casting and carbonization methods, which possess fine thermal management and high-efficiency electromagnetic wave absorption performance [11]. Ma et al. reported wearable silver nanowire decorated leather nanocomposites, with the integrated functions including Joule heating, electromagnetic interference shielding, and piezoresistive sensing [12]. These emerging electromagnetic devices are constantly refreshing people's horizons, vigorously promoting the development of interdisciplinary field.

Advance in electromagnetic and energy devices is inseparable from the research and development of functional materials [13–21]. NiFe_2_O_4_ nanocrystals feature high corrosion resistance and outstanding saturation magnetization along with favorable chemical and thermal stability, with a wide application in functional materials and devices. Meanwhile, in order to improve the electromagnetic response further, rGO nanosheets with excellent dielectric properties are often hybridized with NiFe_2_O_4_ nanocrystals [22–27]. The resulting rGO-NiFe_2_O_4_ displays fine electromagnetic loss and energy attenuation. However, it is regrettable that the charge transport channels in the rGO nanosheets is inevitably destroyed after the introduction of NiFe_2_O_4_ nanocrystals, limiting the dielectric properties to a certain extent.

To address the above problem, an effective molecular patching engineering is proposed [26]. Generally speaking, conductive polymers, featuring stable structure, good flexibility, and high conductivity, can be used to patch destroyed conductivity network. Among them, poly(3,4-ethylenedioxythiophene) (PEDOT) is widely sought after due to its visible transparency, moderate band gap, and fine biocompatibility [28]. However, in order to overcome the poor water solubility, the currently commercialized PEDOT is usually attached to hydrophilic poly(styrenesuifonate) (PSS). The obtained complex achieves a remarkable improvement in hydrophilia, but also inevitably causes a decline in conductivity. More importantly, the precise construction of PEDOT:PSS with shape fidelity and design freedom at nano-micro scale is also very difficult and challenging, which will have an unpredictably effect on the structure and performance of materials and devices.

Oxidative molecular layer deposition (oMLD) is a novel chemical preparation process, allowing precise tailoring of microstructure at micro-nano scale [29]. This process effectively decomposes the traditional chemical gas reaction into two half reactions, which ensures the consistence of the thickness of the grown films [30]. Meanwhile, due to the self-limitation of oMLD process, the prepared films feature good uniformity and high density [31]. More importantly, the oMLD allows the film to grow on complex surface, achieving a uniform coating. Obviously, the high-quality films developed by oMLD present enormous potential in functional materials and advanced devices.

In this work, an eco-mimetic nanoarchitecture is constructed for the first time, successfully achieving the integration of componential and structural advantages to show high-efficiency absorption and attenuation of electromagnetic wave. Its microstructure is controllably tailored by changing the number of oMLD cycles, thereby flexibly tuning electromagnetic properties and internal energy conversion. Through in-depth insight into structure at micro-nano scale, the nature of electromagnetic response is intuitively revealed. On this basis, the frequency-selective microwave absorption is achieved with oMLD, with an optimal reflection loss (RL) up to − 58 dB. Impressively, a novel electromagnetic absorption surface is designed, achieving the ultra-wideband absorption at K and Ka bands. More importantly, an ingenious self-powered energy conversion device is constructed to convert harmful electromagnetic radiation into useful electric energy for recycling, which is very beneficial to the fields of energy and environment. The research results can be generalized toward other electromagnetic functional materials, opening up a new horizon for electromagnetic protection and waste energy recycling.

## Experimental Section

### Materials

Flake graphite powder (grade 325) was purchased from Haida Corporation (Qingdao, China). Sodium nitrate (NaNO_3_), potassium permanganate (KMnO_4_), concentrated sulfuric acid (H_2_SO_4_), hydrogen peroxide (H_2_O_2_), ferric nitrate nonahydrate (Fe(NO_3_)_3_·9H_2_O), nickel nitrate hexahydrate (Ni(NO_3_)_2_·6H_2_O), and ammonium hydroxide (NH_3_·H_2_O) were purchased from Beijing chemical factory (Beijing, China). 3,4-ethoxylenedioxythiophene (EDOT) and molybdenum pentachloride (MoCl_5_) were obtained from Sinopharm Chemical Reagent Co., Ltd. (Beijing, China). All chemical reagents can be directly used without purification.

### Preparation of rGO-NiFe_2_O_4_ (GF) Composite

Graphene oxide (GO) was synthesized from flake graphite by a modified Hummers' method. Typically, 0.5 g of graphite powder, 0.5 g of NaNO_3_, and 23 mL of H_2_SO_4_ were stirred in an ice bath. Meanwhile, 3 g of KMnO_4_ was added slowly; Then, the obtained mixture was stirred for ≈1 h in a water bath (35 ± 5 °C). Next, the mixed solution (100 mL of deionized water and 3 mL of H_2_O_2_) was injected slowly. Finally, the GO was obtained after washing.

The GF composite was prepared by a one-step hydrothermal process. In a typical process, 0.7 mg mL^−1^ of GO suspension was configured. Then, 0.4 mmol of Fe(NO_3_)_3_·9H_2_O and 0.2 mmol of Ni(NO_3_)_2_·6H_2_O were added and stirred to obtain a homogeneous solution. After adjusting pH to 10 by adding dropwise NH_3_·H_2_O, the mixture was transferred to 50 mL of Teflon-lined autoclave and kept at 180 °C for 24 h. Finally, the GF powder was collected by centrifugation, washing, desiccation, and grinding.

### Fabrication of PEDOT Patched GF (P-GF) Eco-Mimetic Nanoarchitecture

P-GF eco-mimetic nanoarchitecture was fabricated by oMLD process in a homemade atomic layer deposition (ALD) reactor. The GF ethanol dispersion was dropped on a quartz substrate, and then was transferred to reactor. The PEDOT was stably assembled on the surface of GF composite by sequential exposure of EDOT monomer (7 s) and MoCl_5_ oxidant (10 s). The reaction temperature is 115 °C. N_2_ purge (60 s) was performed to remove excess raw materials and reaction byproducts. The cycles were executed 20, 40, 60, and 80 times, and the related products were denoted as 20 P-GF, 40 P-GF, 60 P-GF, and 80 P-GF, respectively.

### Materials Characterization

The P-GF eco-mimetic nanoarchitecture was imaged by transmission electron microscopy (TEM) and energy-dispersive x-ray spectroscopy (EDX). The cross section was characterized by atomic force microscopy (AFM) (Bruker, Dimension FastScan). XRD and Raman spectra were recorded by x-ray powder diffractometer (Brucker, D8 Advance) and Raman spectrometer (Renishaw, inVia, 514 nm), respectively. The electrochemical performance was investigated by CHI1660E electrochemical workstation. The complex permittivity and complex permeability (2–18 GHz) were measured by vector network analyzer (Anritsu, 37269D). The RL was calculated based on the measured electromagnetic parameters (Eqs. 1 and 2):

1 $$Z_{{{\text{in}}}} = Z_{0} \sqrt {\frac{{\mu_{r} }}{{\varepsilon_{r} }} } \tanh \left( {j\frac{2\pi fd}{c}\sqrt {\varepsilon_{r} \mu_{r} } } \right)$$

2 $${\text{RL}}\left( {{\text{dB}}} \right) = 20\lg \left| {\frac{{Z_{{{\text{in}}}} - Z_{0} }}{{Z_{{{\text{in}}}} + Z_{0} }}} \right|$$

where *c* is the light velocity, *f* is the frequency of electromagnetic wave, and *d* is the thickness of absorber.

## Results and Discussion

### Fabrication and Characterization of P-GF Eco-Mimetic Nanoarchitecture

**Fig. 1:**
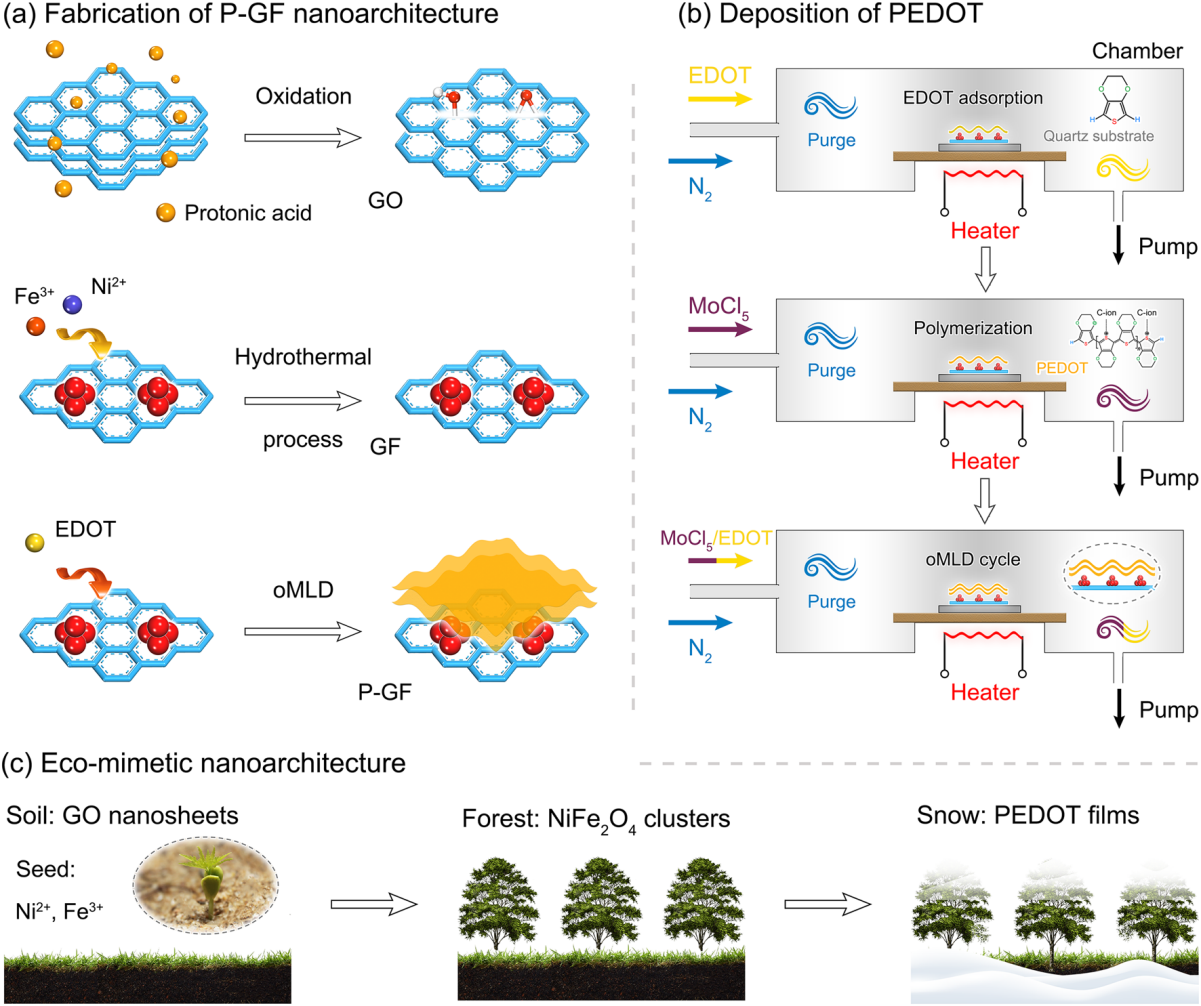
Fabrication and tailoring of P-GF eco-mimetic nanoarchitecture. **a** Illustration of fabrication of P-GF eco-mimetic nanoarchitecture. **b** Schematic illustration of oxidative polymerization of PEDOT by oMLD process (C-ion: Counterion). **c** Inspiration of P-GF eco-mimetic nanoarchitecture from natural environment

Inspired by nature, a novel P-GF eco-mimetic nanoarchitecture is constructed, and the microstructure is precisely tailored by manipulating the thickness of PEDOT films using oMLD process. The preparation and tailoring processes of P-GF eco-mimetic nanoarchitecture are shown in Fig. 1. GO nanosheets chemically prepared from flake graphite possess abundant oxygen-containing functional groups with negative charges (Fig. 1a). Due to the electrostatic interactions, these groups can attract Fe^3+^ and Ni^2+^ with positive charges, along with hydrolysis and nucleation [32]. Meanwhile, the GO nanosheets are reduced, which can inhibit the mass aggregation of NiFe_2_O_4_ nanocrystals. Notably, the C–O–Fe and C–O–Ni linkages possibly form between Fe^3+^/Ni^2+^ and rGO nanosheets. Owing to the magnetic dipole–dipole attraction, the resulting magnetic nanocrystals will grow to form small clusters [33]. After the deposition of PEDOT films, the P-GF eco-mimetic nanoarchitecture is finally obtained.

Figure 1b illustrates the deposition process of the PEDOT films by oMLD process. A complete deposition process is composed of EDOT dosing, N_2_ purge, exposure of MoCl_5_, and N_2_ purge. The prepared GF composite is dispersed into ethanol followed by dropping on a quartz substrate. Then, the substrate is transferred to a homemade ALD reactor after drying in air. The EDOT is heated to 60 °C to obtain reasonable vapor pressure followed by introducing into the reaction chamber. The EDOT vapor can adhered to GF composite by strong physical adsorption (e.g., hydrogen, van der Waals bonding) [30]. N_2_ purge is applied to remove the unadsorbed EDOT. Afterward, MoCl_5_ vapor is introduced and reacted with the adsorbed EDOT. Finally, a N_2_ purge is performed to remove residual gases and byproducts (HCl), and the PEDOT films were obtained. As shown in Fig. 1c, the resulting three-dimensional (3D) P-GF eco-mimetic nanoarchitecture presents a distinct hierarchical design at micro-nano scale, where the graphene, NiFe_2_O_4_ clusters, and PEDOT films can be equivalent to the soil, forest, and snow in nature, respectively.

**Fig. 2:**
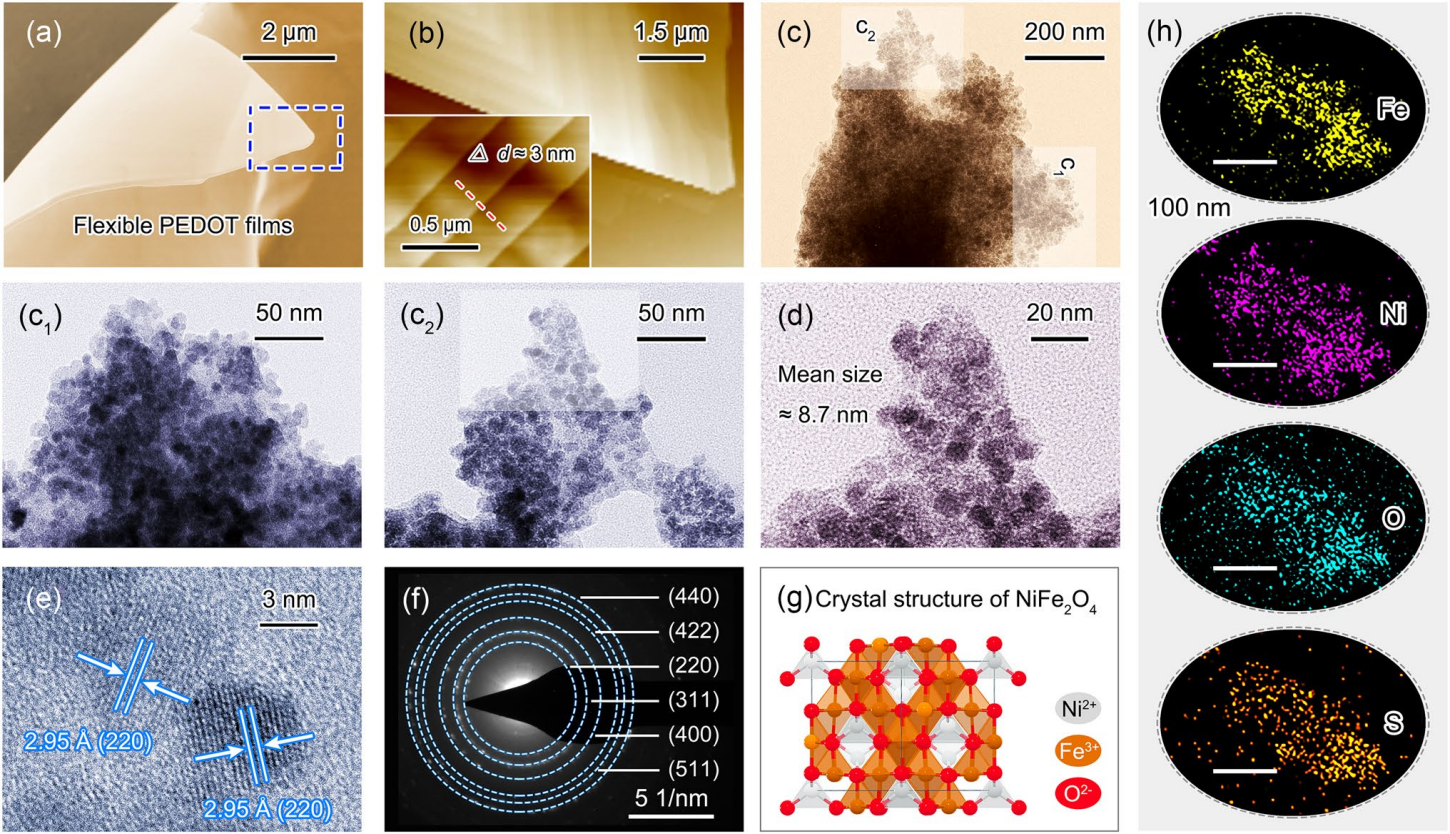
Microstructure characterizations of P-GF eco-mimetic nanoarchitecture. **a** SEM and **b** AFM images of PEDOT films. **c**, **c**_**1**_,** c**_**2**_, and **d** TEM images of P-GF nanoarchitecture. **e** HR-TEM image. **f** SAED pattern of P-GF nanoarchitecture. **g** Crystal structure of NiFe_2_O_4_.** h** Elemental mapping of P-GF nanoarchitecture

The microstructure and morphology of P-GF eco-mimetic nanoarchitecture are observed by SEM, AFM, and TEM images. As shown in Fig. 2a, the prepared PEDOT films feature outstanding flexibility. Further observation by AFM images reveals that the PEDOT films are composed of many secondary nanomembranes with a thickness of 3 nm (Fig. 2b). The P-GF nanoarchitecture is presented by TEM images further, as shown in Fig. 2c, c_1_, c_2_, and d. The magnetic NiFe_2_O_4_ clusters are relatively evenly distributed over rGO nanosheets, and then, the gelatinous PEDOT films are stably coated on the surfaces of GF composites (Figs. S1–S3). The mean size of NiFe_2_O_4_ nanocrystals forming clusters is about 8.7 nm (Fig. 2d). The high-resolution (HR) TEM images are provided to investigate the crystal structure of NiFe_2_O_4_, as shown in Figs. 2e and S3. The spacing of lattice fringe is 2.95 Å, corresponding to (220) crystal plane [34]. Figure 2f is the SAED pattern of NiFe_2_O_4_ nanocrystals, indicating its cubic inverse spinel structure. The corresponding crystal structure model is presented in Fig. 2g. The elemental mapping of P-GF eco-mimetic nanoarchitecture is characterized to investigate its component and microstructure. As shown in Fig. 2h, Fe, Ni, O, and S elements are observed, which confirms the implantation of NiFe_2_O_4_ nanocrystals and the deposition of PEDOT films. Meanwhile, the results also suggest that the P-GF eco-mimetic nanoarchitecture features well-designed hierarchical structure.

### Atomic-Scale Insight into P-GF Eco-Mimetic Nanoarchitecture

**Fig. 3:**
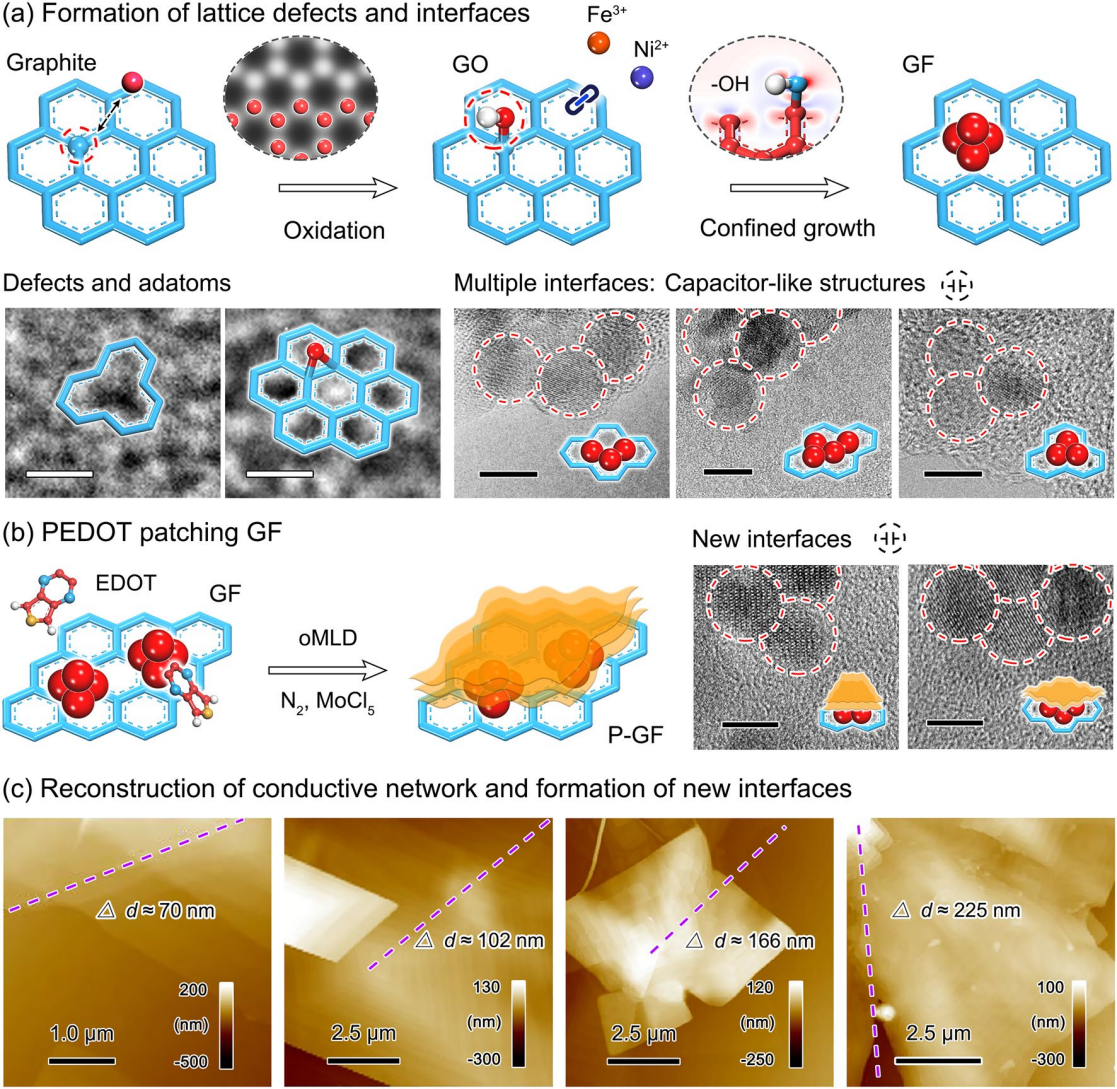
An insight into the microstructure of P-GF eco-mimetic nanoarchitecture. **a** Formation of lattice defects and interfaces, as well as corresponding HR-TEM images. Scale bars are 0.5 nm (defects and adatoms) and 8 nm (multiple interfaces), respectively. **b** Schematic illustration of PEDOT patching GF, and corresponding HR-TEM images. Scale bars are 5 nm. **c** AFM images of P-GF eco-mimetic nanoarchitecture with different thicknesses of PEDOT films. The deposition of PEDOT films reconstructs conductive networks and forms new interfaces

An atomic-scale insight is given to dissect the dielectric genes inside P-GF eco-mimetic nanoarchitecture, as shown in Fig. 3. The prepared GO nanosheets form abundant lattice defects, commonly including Stone–Wales (SW) defects, vacancy defects, large-scale defects (e.g., line defects or planar defects), and oxygen-containing functional groups [35–38]. The SW defect is created by the 90° rotation of C–C bond, not involving the addition or deletion of lattice atoms. The energy barrier of ≈5 eV for reverse transformation guarantees its stability at room temperature after formation. In contrast, the single vacancy (SV) defect arises from the atomic deletion, and is accompanied by the formation of three dangling bonds. Two of the three dangling bonds are apt to the saturation under Jahn–Teller distortion. Due to the low migration barrier, ≈1.3 eV, the SV defects can migrate and coalesce with relative ease, thus constructing double vacancy (DV) defects and even large-scale defects. A point emphasized is that DV defects can also be generated by the continuous deletion of lattice atoms, and can be further saturated to completely remove the dangling bonds (5-8-5 defect). Extrinsic defects form (e.g., oxygen-containing functional groups) when the crystalline order of graphene is perturbed by foreign atoms, namely foreign adatoms (or impurities). The bonding between foreign (noncarbon) atom and graphene determines the effect of atom on the properties of graphene.

After the implantation of magnetic small clusters, the partial lattice defects are reserved for rGO nanosheets (Fig. 3a). Meanwhile, the interfaces equivalent to capacitor-like structures are formed between rGO nanosheets and NiFe_2_O_4_ nanocrystals, and NiFe_2_O_4_ nanocrystals. The differences in size and defect distribution result in the different conductivity (*σ*) or polarity of rGO nanosheets and NiFe_2_O_4_ clusters. Under the electromagnetic field, two sides of the interfaces form different binding charge capacities, which can produce interfacial dipoles.

The implanted NiFe_2_O_4_ clusters introduce additional magnetic response, but also destroy the charge transport channels in the rGO nanosheets. The deposited PEDOT films can patch the GF composites to effectively reconstruct the conductive network inside material system (Fig. 3b). Meanwhile, the plane size of the resulting P-GF eco-mimetic nanoarchitecture is substantially increased from a few microns to tens of microns. Notably, the new interfaces are formed between PEDOT films and NiFe_2_O_4_ clusters, and PEDOT films and rGO nanosheets, which can also form interfacial dipoles under the electromagnetic field. The microstructure of P-GF nanoarchitecture can be controllably tailored by adjusting the thickness of PEDOT films with oMLD process. As observed in Fig. 3c, the thickness of PEDOT films gradually increases with the raised oMLD cycles. The thicknesses of 20 P-GF, 40 P-GF, 60 P-GF, and 80 P-GF samples are about 70, 102, 166, and 225 nm, respectively. Meanwhile, the interfacial dipoles may be formed between secondary nanofilms due to their different size and defect distribution. This tailoring strategy of microstructure based on oMLD process lays a solid foundation for the flexible tuning of electromagnetic properties.

### Electromagnetic Properties of P-GF Eco-Mimetic Nanoarchitecture

**Fig. 4:**
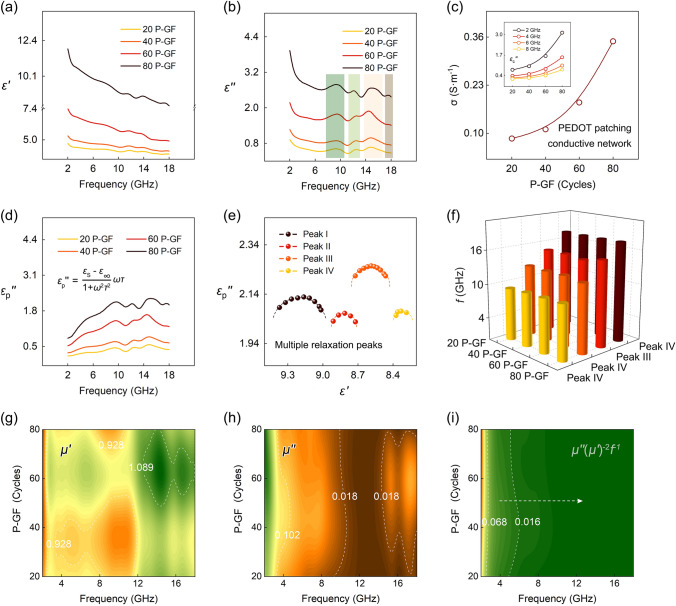
Electromagnetic response of P-GF eco-mimetic nanoarchitecture. Frequency characteristics of **a** *ε*ʹ and **b** *ε*ʺ. **c** Dependance of *σ* and $\varepsilon_{{\text{c}}}^{\prime \prime }$ on oMLD cycles (2, 4, 6, and 8 GHz). **d** Frequency characteristics of $\varepsilon_{p}^{\prime \prime }$. **e** Cole–Cole plots of 80 P-GF nanoarchitecture. **f** Evaluation of center frequency of relaxation peaks. 3D plots of **g** *μ*ʹ, **h** *μ*ʺ, and **i** *μ*ʹ(*μ*ʺ)^−2^*f*^−1^ versus frequency and oMLD cycle

The dielectric response of P-GF eco-mimetic nanoarchitecture is investigated based on the complex permittivity. Figure 4a shows the two-dimensional (2D) plots of real permittivity (*ε*ʹ) versus frequency, where *ε*ʹ stands for the storage capability of electromagnetic energy. The *ε*ʹ gradually drops with the increment of frequency, while it raises as the number of oMLD cycles. Figure 4b shows the imaginary permittivity (*ε*ʺ) of P-GF eco-mimetic nanoarchitecture with different oMLD cycles. Like the *ε*ʹ, the *ε*ʺ decreases with the raised frequency, but increases with the increment of oMLD cycles. The results reveal that the PEDOT patching process can bring stronger dielectric loss to the P-GF eco-mimetic nanoarchitecture. Meanwhile, four relaxation peaks are identified in the plots of *ε*ʺ, which can be indexed to the irregular regions inside P-GF nanoarchitecture, including defects, functional groups, and interfaces. Under an alternating electromagnetic field, the dipoles formed at these structures will break loose, orientate, and produce relaxation losses [26].

According to the Debye theory, the *ε*ʺ depends on the conduction loss ($\varepsilon_{{\text{c}}}^{\prime \prime }$) and relaxation loss ($\varepsilon_{p}^{\prime \prime }$), clarified by the following Eq. 3,

3 $$\varepsilon^{\prime \prime } = \varepsilon_{p}^{\prime \prime } + \varepsilon_{{\text{c}}}^{\prime \prime } = \frac{{\varepsilon_{{\text{S}}} - \varepsilon_{\infty } }}{{1 + \omega^{2} \tau^{2} }}\omega \tau + \frac{\sigma }{{\omega \varepsilon_{0} }}$$

where *ε*_s_ is the static permittivity, *ε*_∞_ is the relative permittivity at high-frequency limit, *ω* = 2π*f* is the angular frequency, and *τ* is the relaxation time.

Figure 4c shows the *σ* of P-GF eco-mimetic nanoarchitecture with different oMLD cycles. The *σ* gradually raises with the increment of oMLD cycles, which can be ascribed to the patching process of PEDOT film to the conductive network of GF composites. The inset shows the 2D plots of $\varepsilon_{{\text{c}}}^{\prime \prime }$ at different EPDOT cycles. As observed, the $\varepsilon_{{\text{c}}}^{\prime \prime }$ increases with lifted oMLD cycles, but decreases with raised frequency, which is consistent with the Debye theory. The frequency characteristics of $\varepsilon_{p}^{\prime \prime }$ are shown in Fig. 4d, and four relaxation peaks can be more intuitively observed (Fig. 4e). Based on our previous work, peaks I-III originate from GF composites, and peak IV is identified to be caused by the PEDOT films [26]. The peak IV of $\varepsilon_{p}^{\prime \prime }$ enhances as the oMLD cycles increase, indicting the accordingly improved contribution of PEDOT films to relaxation loss. Figure 4f depicts the center frequency of all relaxation peaks.

The magnetic response of P-GF eco-mimetic nanoarchitecture is analyzed by real permeability (*μ*ʹ) and imaginary permeability (*μ*ʺ), as shown in Fig. 4g, h. The multiple resonance peaks appear at the 2–18 GHz curves of complex permeability [39]. Among them, the peak with the frequency below 10 GHz is thought to be caused by natural resonance, while peak above 10 GHz is generated by exchange resonance [40]. In addition, the magnetic eddy current is another important source of magnetic loss in the studied frequency band, evaluated by *μ*ʺ(*μ*ʹ)^−2^ *f*^−1^ = 2*πμ*_0_*σd*^2^/3 [41]. Once the *μ*ʺ(*μ*ʹ)^−2^ *f*^−1^ is constant, the magnetic loss of P-GF eco-mimetic nanoarchitecture only depends on magnetic eddy current. Notably, due to the low conductivity of NiFe_2_O_4_ nanocrystals, the eddy current loss is relatively weak (Fig. 4i).

### Microwave Absorption Performance of P-GF Eco-Mimetic Nanoarchitecture

**Fig. 5:**
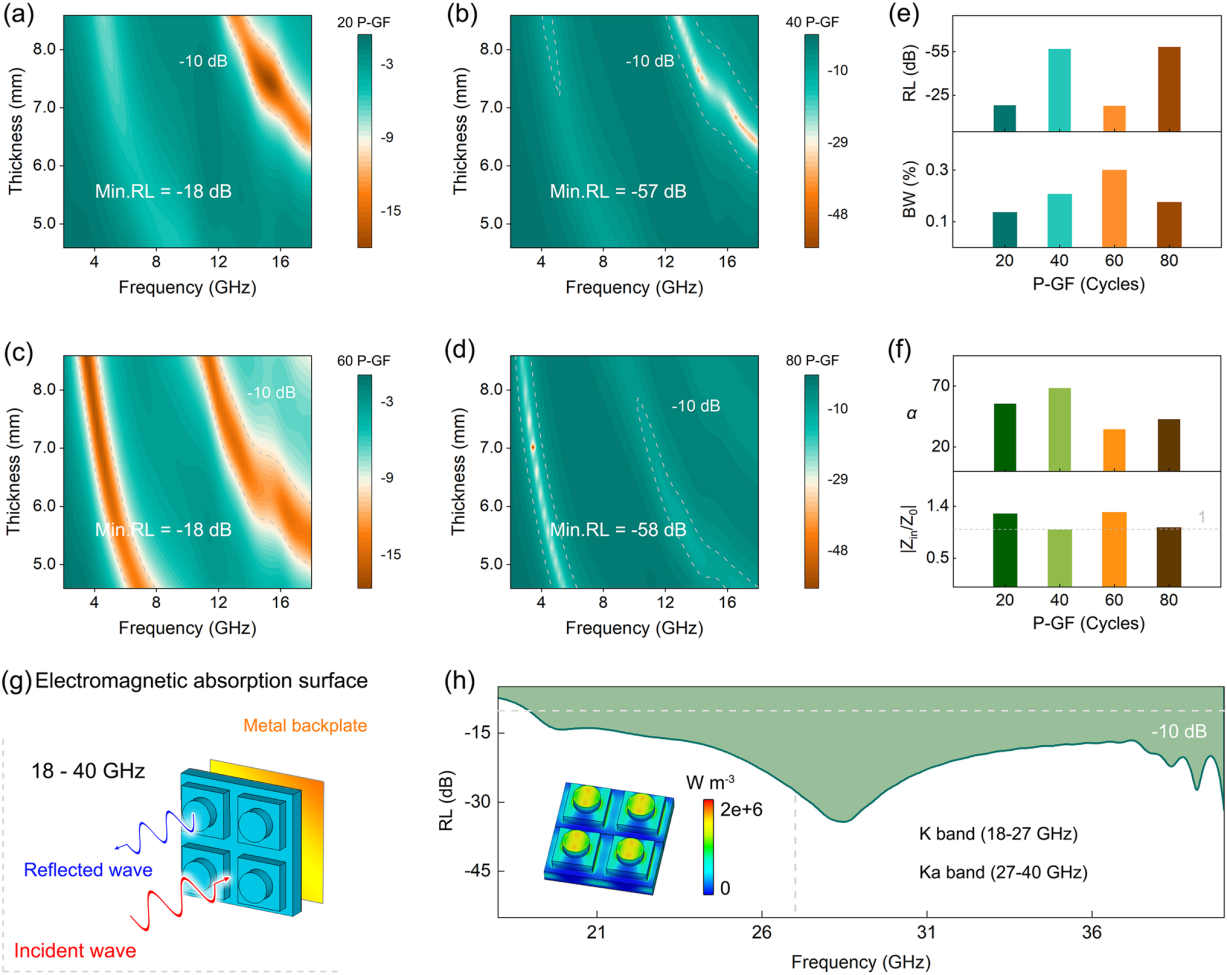
Microwave absorption performance of P-GF eco-mimetic nanoarchitecture. RL of **a** 20 P-GF,** b** 40 P-GF, **c** 60 P-GF, and **d** 80 P-GF. **e** Evaluation of maximum RL (dB) and effective BW (%). **f** Evaluation of *α* and |Z_in_/Z_0_|. **g** Model and **h** absorption performance of high-frequency electromagnetic absorption surface. Inset in **h** is power loss density

Figure 5 presents the microwave absorption performance of P-GF eco-mimetic nanoarchitecture. As shown in Fig. 5a–d, the microwave absorption performance of the eco-mimetic nanoarchitecture can be flexibly tuned by manipulating the number of oMLD cycles. The optimal RL of 20 P-GF nanoarchitecture reaches − 18 dB appearing at 15.12 GHz with an effective bandwidth (BW) (<  − 10 dB) of 2.24 GHz. With oMLD cycles increase to 40 times, the best RL is lifted to − 57 dB (14.08 GHz), and the corresponding − 10 dB BW is up to 3.36 GHz. At the same time, a weak effective absorption peak (the optimal RL <  − 10 dB) appears at 4.88 GHz. The RL of 60 P-GF nanoarchitecture exhibits obvious double absorption peaks. The optimal RL is weakened (only − 18 dB) and shifted from the high-frequency region of the study band to the low-frequency region. When the oMLD cycle reaches 80 times, the optimal RL is enhanced again, reaching − 58 dB at low-frequency 3.44 GHz. At the moment, the effective absorption peak at high frequency tends to disappear.

Figure 5e evaluates the optimal RL and BW (%) of the P-GF eco-mimetic nanoarchitecture. With the increment of the number of oMLD cycles, the thickness of PEDOT films increases gradually. In this process, the optimal RL increases first and then decreases, and the position of the strongest absorption peak is transferred from high frequency to low frequency. The sample of the intermediate state (60 P-GF) possesses the maximum effective BW. The excellent microwave absorption performance highly depends on efficient internal energy attenuation and appropriate impedance matching (|*Z*_in_/*Z*_0_|) [42]. Figure 5f exhibits the attenuation constant (*α*) and |*Z*_in_/*Z*_0_| of the optimal RL of each sample. The good *α* of P-GF eco-mimetic nanoarchitecture is inseparable from the magnetic-dielectric synergy. Meanwhile, the electromagnetic wave absorption performance of absorbers is affected by |*Z*_in_/*Z*_0_|. The |*Z*_in_/*Z*_0_| of ≈1 indicates that the absorbers can maximize the absorption of the incident electromagnetic waves. It can be observed that the appropriate |*Z*_in_/*Z*_0_| (≈1) achieves the highly efficient absorption of 40 P-GF and 80 P-GF to electromagnetic wave, resulting in the best RL.

Based on this, a high-frequency electromagnetic absorption surface is designed using 80 P-GF eco-mimetic nanoarchitecture, allowing the ultra-wideband absorption of incident electromagnetic waves (Fig. 5g, h). The effective absorption BW covers almost the entire K (18–27 GHz) and Ka (27–40 GHz) bands. The minimum RL can reach − 35 dB. This result offers a new idea for high-frequency electromagnetic radiation protection, and simultaneously, suggests that the P-GF eco-mimetic nanoarchitecture is an excellent candidate to construct broadband absorber.

### Self-Powered Energy Conversion Device

**Fig. 6:**
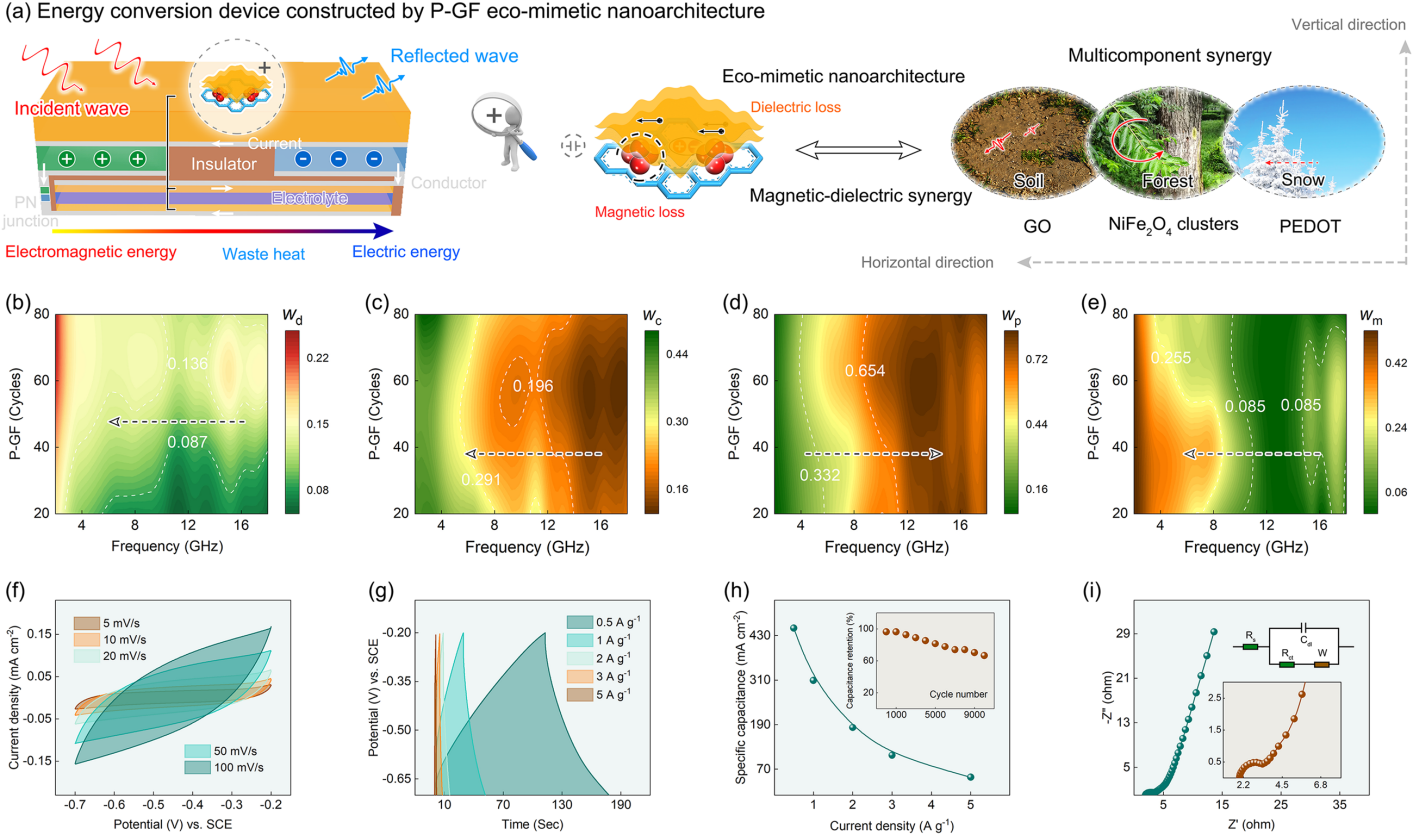
Self-powered energy conversion device.** a** Eco-friendly energy conversion device constructed by P-GF eco-mimetic nanoarchitecture. **b** Electromagnetic energy conversion efficiency inside P-GF eco-mimetic nanoarchitecture. Energy conversion contributed by **c** conduction loss, **d** relaxation loss, and **e** magnetic loss. **f** CV curves of P-GF eco-mimetic nanoarchitecture at different scan rates. **g** GCD curves at different current densities. **h** Specific capacitance and cycling performance.** i** Nyquist plots and corresponding equivalent circuit

Based on energy and environment considerations, a creative self-powered energy conversion device is constructed with P-GF eco-mimetic nanoarchitecture, integrating electromagnetic protection and waste energy recycling. With the help of the device, the harmful electromagnetic radiation is converted into useful electric energy. As shown in Fig. 6a, the device consists of an energy conversion device on the upper layer and an energy storage device on the lower layer. The PN junction is placed in the middle of the two-layer devices to ensure unidirectional transport of the generated current. Among them, the energy conversion device is designed according to the Seebeck effect. The core heat generation module is fabricated with P-GF eco-mimetic nanoarchitecture, which can absorb and convert electromagnetic energy into waste heat. The temperature difference is formed between P-type and N-type semiconductors, and a thermoelectric current is generated. The formed current is eventually stored in the underlying supercapacitor device.

The P-GF eco-mimetic nanoarchitecture, with integrated advantages of structures and components, can efficiently attenuate and convert electromagnetic radiation into waste heat. The rGO nanosheets and PEODT films endow nanoarchitecture with outstanding conduction and relaxation losses, and the magnetic NiFe_2_O_4_ clusters bring about good resonance and eddy current losses. In addition, this is just as the attenuation of electromagnetic signal in the forest after snow is stronger than that of pure soil and vegetation. The P-GF nanoarchitecture mimics the structures and functions of natural environment in vertical and horizontal directions to achieve a high synergy of magnetic and dielectric effects, strongly promoting the highly efficient attenuation of electromagnetic wave. In vertical direction, the P-GF eco-mimetic nanoarchitecture, featuring outstanding hierarchical structure, achieves multiple dissipation of electromagnetic wave. In horizontal direction, the dual-net structure constructed by rGO nanosheets and PEDOT films creates a large surface area, thereby resulting in multiple scattering of electromagnetic wave. Meanwhile, the multi-component synergy of nanoarchitecture further strengthens the attenuation of electromagnetic wave.

The energy conversion efficiency of P-GF eco-mimetic nanoarchitecture is deeply dissected, as shown in Fig. 6b–e. The electromagnetic energy conversion efficiency (*w*_d_) inside P-GF eco-mimetic nanoarchitecture is displayed in Fig. 6b. The *w*_d_ gradually increases with the thickness of PEDOT films, but continuously decreases with the frequency. On this basis, the energy conversion efficiency contributed by conduction loss (*w*_c_), relaxation loss (*w*_p_), and magnetic loss (*w*_m_) is further explored. As shown in Fig. 6c–e. The relaxation loss dominates the attenuation and conversion of high-frequency electromagnetic energy in the study band, while the conduction loss and magnetic loss determine the conversion of low-frequency electromagnetic energy [26]. Notably, due to the effect of exchange resonance, the maximum value of the *w*_p_ appears at ≈ 13 GHz.

In order to simplify process and reduce costs, the supercapacitor is also fabricated with P-GF eco-mimetic nanoarchitecture, and the related electrochemical performance is investigated, as shown in Fig. 6f–i. The cyclic voltammetry (CV) curves of P-GF eco-mimetic nanoarchitecture show no obvious redox peaks, indicating the weak contribution of redox reactions to performance (Fig. 6f). Figure 6g shows the galvanostatic charge–discharge (GCD) curves with a potential range of − 0.7 to − 0.2 V, which is applied to evaluate storage performance. The intuitive results are presented in Fig. 6h. When the current density is 1 A g^−1^, the specific capacitance reaches 450 mA cm^−2^. After 10,000 cycles, the capacitance retention (%) still exceeds 65%. The electrochemical impedance spectroscopy (EIS) of P-GF eco-mimetic nanoarchitecture is shown in Fig. 6i. The equivalent series resistance (*R*_s_) and charge transfer resistance (*R*_ct_) are 1.98 Ω and 0.94 Ω, respectively. Undoubtedly, the creative energy conversion device is expected to serve as space operations, interplanetary exploration, long-range transportation, and others in the future.

## Conclusion

In summary, drawing wisdom and inspiration from nature, an eco-mimetic nanoarchitecture is constructed for the first time, featuring excellent electromagnetic response. The oMLD process is applied to tailor the microstructure, achieving tunable absorption and attenuation of electromagnetic wave. The optimal RL reaches − 58 dB, and the absorption frequency can be manipulated from high frequency to low frequency. The insight at the micro-nano scale reveals the unique advantages of the nanoarchitecture in energy loss and conversion, including magnetic-dielectric synergy, multiple scattering, impedance matching optimization, and others. Furthermore, a high-frequency electromagnetic absorption surface is designed, allowing ultra-wideband absorption ranging from K to Ka bands. More impressively, a creative self-powered energy conversion device is constructed with P-GF eco-mimetic nanoarchitecture to absorb and convert harmful electromagnetic radiation to useful electric energy for recycling, offering a new horizon for electromagnetic protection and waste energy recycling. Undoubtedly, this work powerfully promotes the development of novel electromagnetic functional materials and devices, which shows great promise toward applications in electromagnetic protection, intelligent robot, virtual reality, spaceship, etc.

### Supplementary Information

Below is the link to the electronic supplementary material.

Supplementary file1 (PDF 292 KB)
