# Supplementary material for: Oxidative Molecular Layer Deposition Tailoring Eco-Mimetic Nanoarchitecture to Manipulate Electromagnetic Attenuation and Self-Powered Energy Conversion
Source: Nanomicro Lett. 2023 May 31;15:142. doi: 10.1007/s40820-023-01112-7 (PMC10232706; doi:10.1007/s40820-023-01112-7)
Supplement: Supplementary file 1 — Supplementary file1 (PDF 292 KB) [file 40820_2023_1112_MOESM1_ESM.pdf]

Supporting Information

Oxidative Molecular Layer Deposition Tailoring Eco-Mimetic Nanoarchitecture to Manipulate Electromagnetic Attenuation and Self-Powered Energy Conversion

Jin-Cheng Shu[1], Yan-Lan Zhang[1], Yong Qin[2], Mao-Sheng Cao[1, *]

[1]School of Materials Science and Engineering, Beijing Institute of Technology, Beijing 100081, P. R. China

[2]Chinese Acad Sci, Inst Coal Chem, State Key Lab Coal Convers, 27 Taoyuan South Rd, Taiyuan 030001, Shanxi, P. R. China

*Corresponding author. E-mail: caomaosheng@bit.edu.cn (Mao-Sheng Cao)

Supplementary Figures

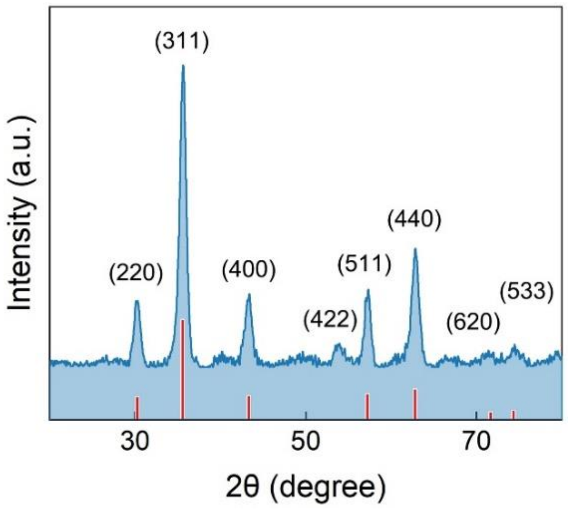

Fig. S1 XRD pattern of GF composite

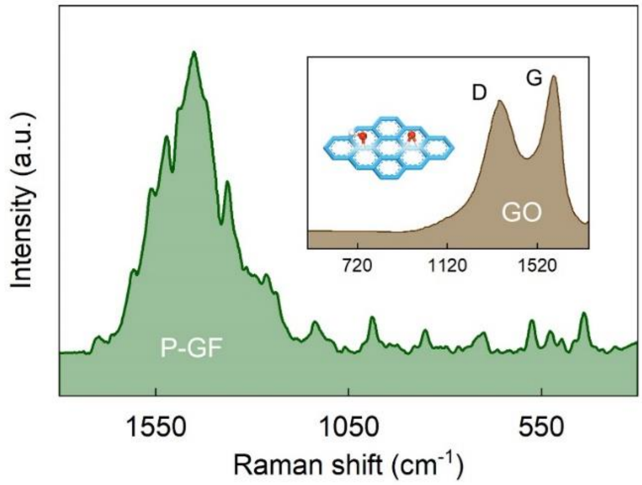

Fig. S2 Raman spectrum of P-GF eco-mimetic nanoarchitecture. Inset is the Raman spectrum and model of GO

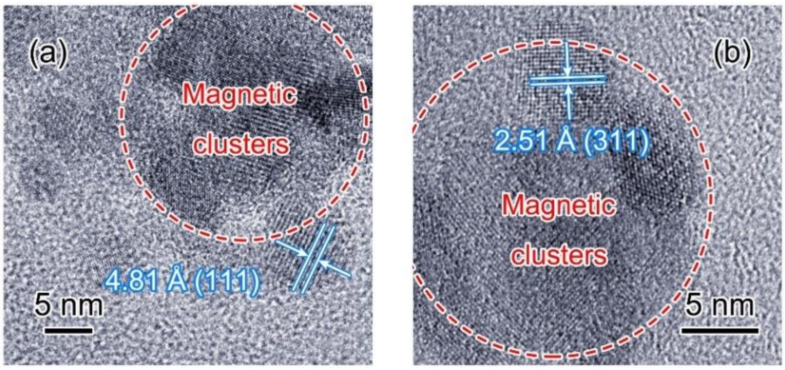

Fig. S3 HR-TEM images of P-GF eco-mimetic nanoarchitecture
